# Effects of pine wilt disease invasion on soil properties and Masson pine forest communities in the Three Gorges reservoir region, China

**DOI:** 10.1002/ece3.1326

**Published:** 2015-03-25

**Authors:** Ruihe Gao, Juan Shi, Ruifen Huang, Zhuang Wang, Youqing Luo

**Affiliations:** Beijing Key Laboratory for Forest Pest Control, Beijing Forestry UniversityBeijing, 100083, China

**Keywords:** *Bursaphelenchus xylophilus*, invasive alien species, Masson pine, pine wilt disease, soil property, three Gorges reservoir region

## Abstract

Pine wilt disease (PWD) has caused significant Masson pine mortality in the Three Gorges reservoir region in central China. In this study, five uniform Masson pine stand types infected by PWD were selected and surveyed on slopes and aspects with similar environmental conditions. In sites that had been infected, soil bulk density was reduced, and the difference among the groups was statistically significant (*P *<* *0.05) at the 0–10 cm and 10–20 cm soil layers, but not at 20–40 cm. Other soil water-related physical properties, excluding noncapillary porosity, significantly differed among the groups in all soil layers. Additionally, the values of available phosphorus, sodium, potassium, calcium, and magnesium were higher in the invaded stands, but the total nitrogen and organic matter contents were lower. Masson pine does not become reestablished following PWD-induced mortality but is instead replaced by broad-leaved tree species. Among the 19 examined environmental variables, five were found to be significantly related with the ordination of plant community structure: Masson pine stumps (MPS), K^+^, capillary water holding capacity (CWHC), capillary porosity (CP), and soil water content (SWC). Among these factors, the plant community structure was principally related to MPS and K^+^. The findings of this study show that the outbreak of PWD has impacted Masson pine forest soil properties and altered forest community composition. The disease is negatively related with the presence of Masson pine and positively associated with that of broad-leaved tree species.

## Introduction

**Figure 1:**
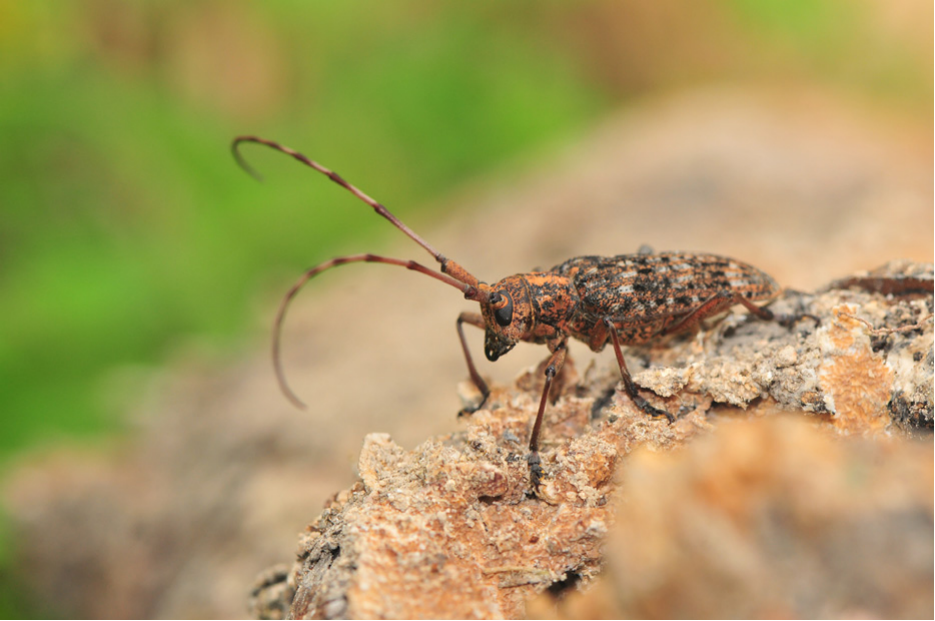
Pine sawyer beetle (*Monochamus alternatus* Hope) is one of the most important vector of pine wood nematode in China.

With the rapid development of international trade, tourism, and transportation, invasive alien species have become increasingly common and pose a great threat to regional biodiversity, ecosystems, and human health (Shi et al. 2010). Invasive alien species have triggered dramatic declines in ecologically important forest tree species, resulting in enormous environmental impacts, the disruption of ecosystem processes, and decreased native species genetic diversity and abundance, as well as variation in population structure and forest community composition (Cohen and Carlton 1998; Curnutt 2000; Ellstrand and Schierenbeck 2000; Daehler and Carino 2001; Hejda et al. 2009; Gandhi and Herms 2010; Hulcr and Dunn 2011). Approximately 488 nonindigenous species are now established in China, including 171 animals, 265 plants, 26 fungi, 3 protists, 11 prokaryotes, and 12 viruses (Xu et al. 2012). The pine wood nematode *Bursaphelenchus xylophilus* (Steiner and Buhrer) Nickle (Nematoda: Aphelenchoididae), which originated in North America and causes the destructive pine wilt disease (PWD) (Dropkin et al. 1981; Shi et al. 2007), is identified as the leading forest pest in China (Wan et al. 2005; Shi et al. 2006). Symptoms of PWD include the loss of green color in infected needles, turning them reddish brown while retaining them on the tree (Dropkin et al. 1981). *Bursaphelenchus xylophilus* can be transferred from infected pine trees to healthy ones via its vector, the pine sawyer beetle (*Monochamus alternatus* Hope (Fig.1)). Once infected, Masson pines (*Pinus massoniana* Lamb.) die within 2–3 months (Hu et al. 2012), and no effective biological or chemical control policies currently exist for PWD in forest ecosystems.

The Three Gorges Project is one of the largest hydropower complex projects in the world. This multiobjective development project has great potential benefits for flood control, power generation, and navigation. The Three Gorges reservoir region is located at the north rim of the mid-subtropical region of central China, where the climate becomes more temperate. The varied topography of the Three Gorges reservoir region provides a sensitive ecological transition zone for its fauna and flora. Masson pine, which is an indispensable forest species in the Three Gorges reservoir region, is widely distributed across 19 southern provinces in China and accounts for approximately 10% of the national total area of forest resources (Chen et al. 1997). The pine is a pioneer tree species for afforestation in South China and plays an important role in wood production, C sequestration, and the provision of ecosystem services (Wilson 1993; Zhang et al. 2006b). Of even greater concern is the severe harm that may occur to these forests by the serious invasion of *B. xylophilus*. Therefore, PWD poses an extremely grave threat to the 5.69 × 10^5^ ha^2^ of Masson pine forest in the Three Gorges reservoir region, and related problems such as soil erosion, landslides, and high rates of sediment accumulation threaten the ecological security of the Three Gorges Dam.

**Figure 2:**
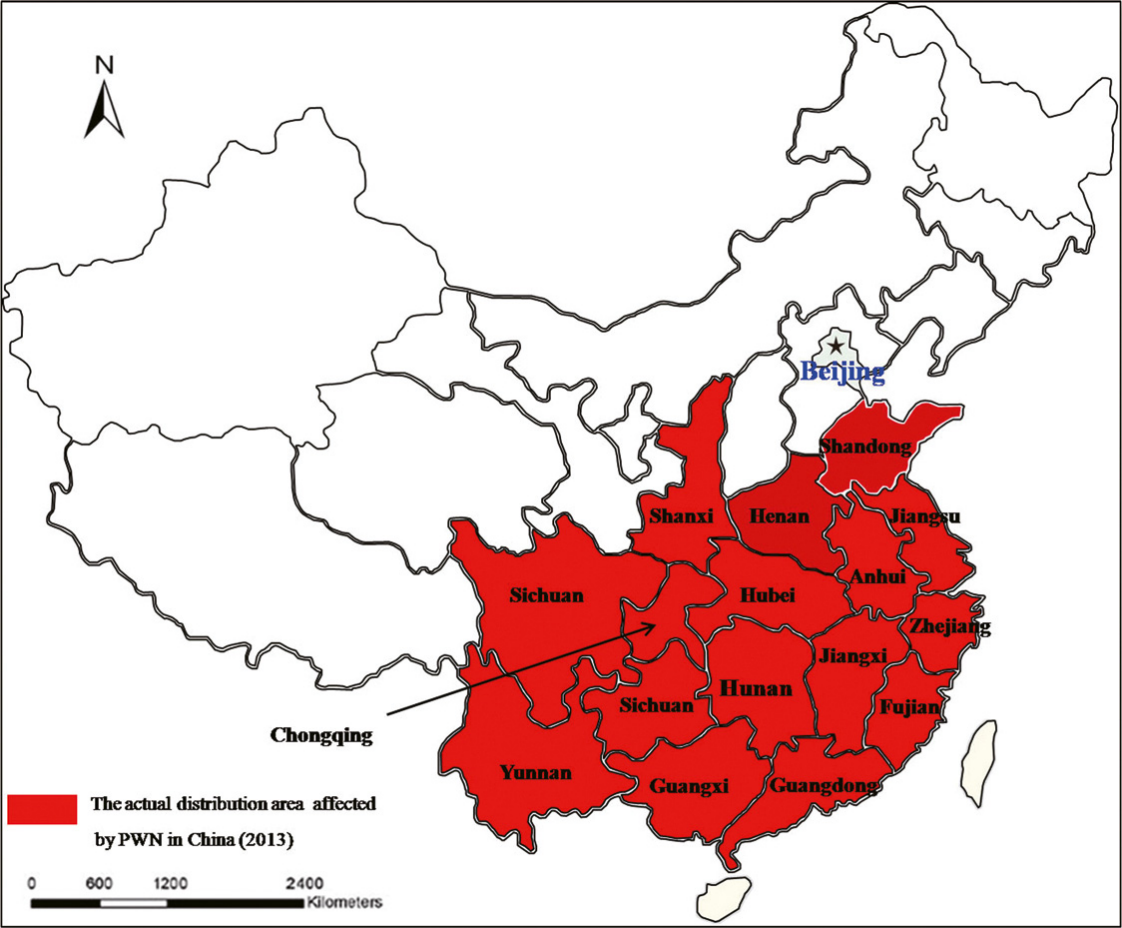
Current distribution of pine wilt disease throughout the southeastern provinces of China (2013). (Data obtained from the No.2 bulletin of State Forestry Administration in 2014—The epidemic area of Pine Wilt Disease. www.forestry.gov.cn/main/3600/content-657977.html).

In China, PWD was first detected in 1982 at Sun Yat-Sen's Mausoleum in Nanjing. By the end of 2013, this disease had spread to 16 provinces and 178 counties (Fig.2) (The bulletin of State Forestry Administration 2014). Unfortunately, PWD affects several economic and ecological processes on multiple scales; cutting down a tract of damaged pine trees causes a serious economic loss of forest resources, alters the forest microenvironment, modifies the composition of woodland plant and insect species, and alters the landscape structure (Shi et al. 2007, 2013; Gao et al. 2013). Recommendations for the control of this disease have focused on the direct control of pine bark beetles by felling, debarking, and immediately burning damaged pines (Suzuki 2002). Therefore, the invasion of *B. xylophilus* has caused considerable economic losses to the timber value and economic value of pine forests (Yu et al. 2011). Moreover, the risk of the spread of PWD is increasing due to the high frequency of trade in the global economy, the active domestic economy, and the implementation of several national basic construction projects (Xie et al. 2009; Shi et al. 2013).

Numerous studies are required to devise an effective control system for PWD in affected regions and to prevent its rapid spread. Previous studies of PWD have mainly focused on pine sawyer beetles, host plants, morphological analysis, and molecular biological examination of *B. xylophilus*, etc. (Mamiya 1988; Yoshimura et al. 1999; Gao et al. 2013; Shi et al. 2013). Other studies have examined the relationship between PWD and various environmental factors and soil properties, such as temperature (Mamiya 1988; Yoshimura et al. 1999), light conditions (Mabuhay and Nakagoshi 2012), soil water content (Suzuki and Kiyohara 1978; Miki et al. 2001; Akema and Futai 2005), and soil physicochemical properties (Kim et al. 2010; Mabuhay and Nakagoshi 2012). Environmental factors influence the spread of PWD (Mabuhay and Nakagoshi 2012), and soil conditions can be altered when drastic variations of forest structure occur due to the invasion of *B. xylophilus*. Research into the changes of soil properties is critical for understanding the ecological consequences of vegetation recovery (Makeschin 1994; Paniagua et al. 1999). Yet, how the effects of PWD on forest plant community structure and soil properties vary depending on the invasion of the disease remains relatively unexplored. Therefore, it is vital to examine the changes in soil conditions in forest stands that have suffered infection by PWD.

Biological disturbances due to exotic pathogens and insects can result in the selective loss and replacement of particular tree species, causing significant changes to ecosystem composition and processes (Castello et al. 1995; Spiegel and Leege 2013). These disturbance effects can be seen at the forest and ecosystem levels; initial changes in tree species composition then affect ecosystem characteristics such as forest structure, productivity, nutrient cycling, soil organic production and turnover, hydrology, and the food web (Lovett et al. 2006). The rapid tree death caused by PWD is likely to cause serious biological disturbances in areas where pine trees were once abundant, because the mortality of pine trees may significantly impact plant population structure, canopy creation, and forest community composition. Several stand-level characteristics change during forest vegetation succession after PWD infection, including light and soil conditions, tree population structure, and the forest community.

The objective of the current study was to investigate the dynamic changes of soil properties and the composition and distribution of plant community structure in various Masson pine forest stands damaged by PWD. We addressed the following questions for damaged Masson pine forest sites in the Three Gorges reservoir region: (1) What are the effects of Masson pine mortality on soil properties? (2) What are the effects of Masson pine mortality on forest composition and community structure? (3) What is the relative importance of different environmental variables in structuring plant populations after infection by the pine wood nematode?

## Materials and Methods

### Study sites

**Figure 3:**
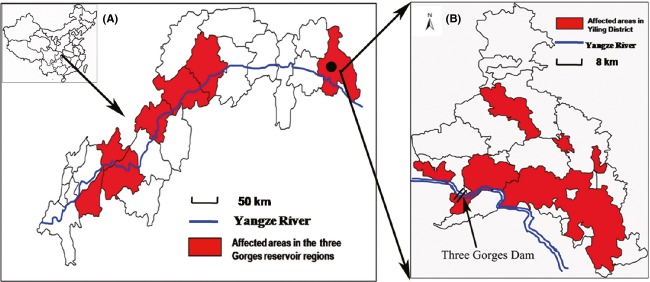
The location of Yiling District (study areas), Hubei province, P.R. China. (A) Current distribution of pine wilt disease throughout the counties in the Three Gorges reservoir regions (2013); (B) Current distribution of pine wilt disease areas in Yiling District. (Data obtained from the Station of Pest and Disease Control and Quarantine of Yiling District, Hubei province, P.R. China).

The study sites were located in the Yiling District (latitude 30°32′–31°28′N, longitude 110°51′–111°39′E), an eastern part of the Three Gorges reservoir region containing the demarcation point of the upper and middle reaches of the Yangtze River (Fig.3). This area has an eastern mid-subtropical monsoon climate, with a mean annual precipitation and mean annual temperature of 997–1370 mm and 16.6°C, respectively. Masson pine trees are distributed widely in this region at an altitude of 45–1500 m, reaching from the bank of the Yangtze River to the top of the mountain. *Bursaphelenchus xylophilus* attacked the Masson pines in the Yiling District in 2006; PWD has since spread rapidly, reaching its peak in 2012.

Fifteen Masson pine plots were established with loamy sand soil, the same bedrock type, and same-facing slopes and aspects to minimize spatial variations in the soil properties (Kim et al. 2010). The plots were divided into five stand types: ST1 was an uninfected control, while ST2, ST3, ST4, and ST5 had all been infected by PWD and contained 2.67, 4.33, 6.67, and 8.50 Masson pine stumps per 100 m^2^, respectively. Three permanent 30 m × 30 m Masson pine forest plots were established on separate sites for each of the stand types in this study (Table1). Selective cutting of infected and dead pine trees was conducted every winter after the appearance of PWD in the infected Masson pine forest plots.

**Table 1:** Stand characteristics of five Masson pine forest sites infected by pine wood nematode

Stand	ST1			ST2			ST3			ST4			ST5		
	Total	*P. massoniana*	Other spp.	Total	*P. massoniana*	Other spp.	Total	*P. massoniana*	Other spp.	Total	*P. massoniana*	Other spp.	Total	*P. massoniana*	Other spp.
Number of stems (tree ha^−1^)	1836 ± 205.51	1303 ± 174.62	533 ± 65.11	1489 ± 226.90	933 ± 171.05	556 ± 117.06	1304 ± 289.17	704 ± 243.77	600 ± 155.56	1263 ± 281.38	537 ± 162.49	726 ± 145.01	1141 ± 150.03	348 ± 203.77	793 ± 72.86
Mean DBH^1^ (cm)	9.22 ± 1.26	10.79 ± 1.41	4.16 ± 0.42	9.63 ± 1.21	12.58 ± 1.77	4.67 ± 0.12	10.51 ± 0.72	13.83 ± 0.42	6.62 ± 1.28	9.95 ± 2.25	15.03 ± 2.53	6.18 ± 2.10	15.43 ± 0.97	21.24 ± 1.99	12.17 ± 0.93
Basal area (m^2^/ha)	16.29 ± 2.41	15.54 ± 2.62	0.75 ± 0.47	14.65 ± 1.18	13.57 ± 1.34	1.09 ± 0.16	14.96 ± 3.93	12.38 ± 4.65	2.58 ± 0.74	14.92 ± 7.36	11.96 ± 4.73	2.96 ± 0.92	21.78 ± 4.45	13.09 ± 5.53	8.69 ± 1.24
Soil texture (sand:silt:clay)	Loamy sand (87:5:8)			Loamy sand (84:5:11)			Loamy sand (84:6:10)			Loamy sand (83:8:9)			Loamy sand (80:9:11)		
Masson pine stumps (100 m^2^)	0			2.67			4.33			6.67			8.5		
Elevation (m)	141.00 ± 41.39			211.67 ± 71.32			214.33 ± 83.20			232.67 ± 77.89			223.33 ± 41.79		
Slope (^°^)	24.00 ± 2.65			18.33 ± 4.16			23.32 ± 7.76			21.00 ± 5.57			17.33 ± 4.51		

1 DBH, diameter at breast height.

### Field surveys

Field surveys were conducted in 12 infested plots and three control plots to collect tree data for all trees with a diameter at breast height (DBH) ≥ 2.5 cm, including species name, tree height, DBH, and status (live or dead). In addition, environmental factors such as altitude and slope were recorded. As infected Masson pine trees had been cut down in the infected plots, the quantity of cut stumps was measured to calculate the damage rate.

### Soil sample collection and processing

Soil samples were collected for physical and chemical analyses by digging soil pits at five randomly selected points in each plot. Using the cutting ring method (ring = 100 cm^3^), soil samples were collected at 0–10 cm, 10–20 cm, and 20–40 cm in depth and then taken to the laboratory to be measured for water-related physical properties. Soil particle size distribution was determined through a hydrometer method. Soil bulk density (BD, g/cm^3^) samples were dried at 105°C to measure bulk density. The soil water content (SWC, %) was measured from the mass loss after oven drying the samples at 105°C for 48 h to a constant weight. The natural state of the soils, along with the cutting rings, was weighed (*m*_1_, g) after soaking for 12 h in water to estimate the maximum water holding capacity (MWHC, %). The cutting rings were then placed on dry sand for 2 h, allowing the nonpore water to be completely drained, then weighed (*m*_2_, g) and used to calculate the capillary water holding capacity (CWHC, %). Finally, the soil was sampled from the cutting rings and dried in an aluminum box to a constant weight (*m*_0_, g). The equations of maximum water holding capacity, capillary water holding capacity, capillary porosity (CP, %), noncapillary porosity (NP, %), and total porosity (TP, %) were as follow (Zhang and Xu 1986; Zhang et al. 2006a):

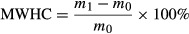
1

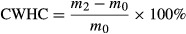
2

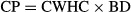
3

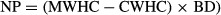
4

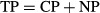
5

For chemical analyses, soil samples were collected from 0–40 cm in depth and then mixed and homogenized; rocks, litter, and other large particles were removed manually. The soil was then air-dried, ground, and passed through a 2 mm sieve. Subsamples of soil were stored at 4°C until use. The soil pH level was tested in a 1:5 mixture of soil:DI water using a glass electrode. Soil organic matter (%) was analyzed using the potassium dichromate oxidation method. Total nitrogen (%) was determined using the Kjeldahl nitrogen method. Available phosphorus (%) was monitored with a UV-Vis spectrophotometer (UV-2550, Shimadzu, Kyoto, Japan). Subsamples of soil were analyzed for potassium (%), sodium (%), calcium (%), and magnesium (%) using an atomic absorption spectrophotometer (SpectrAA220, Varian, Australia).

### Importance values

Relative density, relative frequency, and relative dominance were summed using the basal area of trees ≥ 2.5 cm in DBH to calculate the importance value (IV) for each woody species in the five stand types. The following formula was used to calculate the IV for each tree layer (Spiegel and Leege 2013):

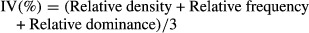
6

where relative density = (absolute density for species i/∑ of density for all species) ×100; relative frequency = (absolute frequency for species i/∑ of freq. for all species) ×100; relative dominance = (absolute dominance for species i/∑ of dom. for all species) ×100.

### Data analysis

Analysis of covariance (ANCOVA) was conducted among the means of the soil chemical and physical parameters in the different stand types. The least significant difference (LSD) test at the *P *<* *0.05 level was used to compare the means of the soil parameters when the results of the ANOVA were significant at the *P *<* *0.05 level. All of the statistical analyses were performed using SPSS 18.0 for Windows, SPSS Inc., Chicago, IL, USA.

The variation in plant or animal populations along a series of environmental variables can be analyzed through the method of ordination (Lepš and Šmilauer 2003). Redundancy analysis (RDA) is a constrained linear form of principal component analysis based on Euclidean distance (Legendre and Legendre 2012). This method provides a direct multivariate statistical tool for identifying the particular factors that influence the composition of community structure among large sets of environmental variables. Relatively few studies have used RDA to relate environmental variables to the distribution of plant or insect community structure in pine forests damaged by PWD. Gao et al. (2013) used woodland environmental variables to analyze the distribution of parasitic insects under different environmental gradients in a Masson pine forest damaged by PWD at Zhoushan Island, Zhejiang Province, in eastern China.

The ordination of the plant community structure among the different stand types was determined using CANOCO 5.0 (Microcomputer Power, Ithaca, NY) according to Ter Braak and Šmilauer (2012). The plant species data (quantity of each tree species, IV > 1.00) were subjected to logarithm transitions and used as individual response variables. The largest gradient length obtained using detrended correspondence analysis (DCA) was 1.45; therefore, RDA was the most appropriate analytical method (Lepš and Šmilauer 2003). Based on the field survey and biological measurements, a total of 19 environmental variables were employed in the analysis of plant community structure, including the quantity of Masson pine stumps, slope, altitude, canopy closure, and soil physical and chemical properties (from 0 to 40 cm in depth) of each study site. Based on a Monte Carlo permutation test with 499 iterations, the forward selection procedure was used to determine the most significant environmental variables (*P < *0.05) for the ordination of plant community structure, and the selected significant variables were used in the RDA (You et al. 2014).

## Results

### Dynamic changes in soil water-related physical properties and chemical properties

The soil water-related physical properties varied greatly across the stand types (Table2). At sites with more broad-leaved tree species, there was a nonsignificant trend toward decreased bulk density. Compared with the control stands (ST1), the value of soil bulk density at the infected stands (ST2, ST3, ST4, and ST5) was lower, and the differences between the groups were statistically significant (*P *<* *0.05) at the 0–10 cm and 10–20 cm soil layers. At sites with *B. xylophilus* infestations, total porosity and capillary porosity were higher in all soil layers (*P *<* *0.05). However, no significant differences were observed for soil noncapillary porosity (*P *>* *0.05). The results also showed that maximum water holding capacity, capillary water holding capacity, and soil water content were higher at the infected sites than at the control sites, and these differences between stand types were significant for all soil layers (*P *<* *0.05).

**Table 2:** Soil water-related physical properties after different periods of infection by pine wood nematode

Soil layer (cm)	Stand type	Bulk density (g/cm^3^)	Maximum water holding capacity (%)	Capillary water holding capacity (%)	Noncapillary porosity (%)	Capillary porosity (%)	Total soil porosity (%)	Soil water content (%)
0–10 cm	ST1	1.50 ± 0.10a	25.25 ± 2.68a	20.17 ± 1.48a	3.42 ± 1.52a	13.53 ± 1.51a	16.95 ± 2.44a	14.19 ± 2.57a
	ST2	1.49 ± 0.022a	30.13 ± 3.34ab	24.90 ± 2.10a	3.49 ± 1.64a	16.61 ± 1.66ab	20.10 ± 2.46ab	16.66 ± 1.49a
	ST3	1.33 ± 0.185b	32.85 ± 5.81b	24.97 ± 3.50a	6.88 ± 2.58	19.57 ± 4.60b	26.46 ± 7.15bc	14.09 ± 3.36a
	ST4	1.28 ± 0.093b	40.27 ± 7.49c	34.84 ± 7.36b	4.29 ± 3.02a	27.67 ± 7.58c	31.96 ± 8.26c	29.63 ± 9.74b
	ST5	1.39 ± 0.122ab	33.38 ± 4.27b	23.85 ± 2.98a	6.98 ± 4.13a	17.31 ± 3.28ab	24.29 ± 4.97b	17.75 ± 2.75a
	*F*-value	4.247**	7.103**	10.852**	2.508	9.064**	6.442**	10.155**
	*P*-value	0.009	0.001	0.000	0.068	0.000	0.001	0.000
10–20 cm	ST1	1.61 ± 0.078a	25.31 ± 3.82a	21.32 ± 3.66a	2.50 ± 1.64a	13.38 ± 2.86a	15.88 ± 3.08a	16.39 ± 3.93a
	ST2	1.43 ± 0.102ab	30.89 ± 4.26ab	23.88 ± 1.76ab	5.06 ± 3.43a	16.85 ± 2.21ac	21.91 ± 4.75ab	16.72 ± 2.54a
	ST3	1.49 ± 0.156ab	27.52 ± 5.19a	20.91 ± 4.38a	4.56 ± 2.24a	14.29 ± 4.38a	18.85 ± 5.64ab	14.36 ± 3.66a
	ST4	1.37 ± 0.126b	36.64 ± 6.94b	34.56 ± 7.13c	3.45 ± 0.60a	25.74 ± 7.17bc	27.19 ± 7.22a	30.92 ± 8.42b
	ST5	1.42 ± 0.074b	34.26 ± 4.14b	29.31 ± 5.78bc	3.47 ± 1.99a	20.77 ± 4.87c	24.24 ± 4.04a	24.21 ± 6.35c
	*F*-value	3.935*	5.523**	8.539**	2.755	7.253**	4.394**	11.232**
	*P*-value	0.013	0.003	0.000	0.050	0.001	0.008	0.000
20–40 cm	ST1	1.55 ± 0.071a	28.05 ± 4.11a	22.24 ± 5.78a	3.79 ± 3.04a	14.4 ± 3.99a	18.19 ± 3.24a	18.35 ± 5.51a
	ST2	1.43 ± 0.023a	28.33 ± 1.05a	21.38 ± 3.32a	4.89 ± 2.69a	14.93 ± 2.20a	19.82 ± 1.06a	17.72 ± 2.76a
	ST3	1.52 ± 0.243a	28.81 ± 7.92a	23.13 ± 6.58a	4.01 ± 2.55a	16.04 ± 6.08a	20.05 ± 7.97a	14.70 ± 5.30a
	ST4	1.37 ± 0.107a	37.01 ± 5.45b	33.77 ± 3.03b	2.53 ± 3.64a	24.83 ± 3.29b	27.36 ± 6.15b	30.52 ± 3.73b
	ST5	1.37 ± 0.076a	35.53 ± 4.34b	31.81 ± 3.65b	2.75 ± 0.90a	23.37 ± 3.47b	26.12 ± 4.13b	26.41 ± 1.79b
	*F*-value	2.538	4.398**	9.325**	0.747	9.065**	3.966*	17.609**
	*P*-value	0.065	0.008	0.000	0.569	0.000	0.013	0.000

Values are mean ± SD. For each column, values with different letters are significantly different at *P *=* *0.05.

* *P *<* *0.05;

** *P *<* *0.01.

By contrast, the soil chemical properties were not significantly different among the different stand types (*P *>* *0.05, Table3). The soil pH was slightly acidic and showed little fluctuation (range from 4.78 to 6.89), with no significant differences observed between the groups. In the infected stands, the levels of available phosphorus, sodium, potassium, calcium, and magnesium were slightly higher than in the control stand. However, the opposite trend was observed for total nitrogen and organic matter.

**Table 3:** Soil chemical properties after different periods of infection by pine wood nematode

Stand type	Total *N* (%)	Organic matter (%)	Soil pH (5:1)	Available P (mg/kg)	K^+^ (mg/kg)	Na^+^ (mg/kg)	Ca^2+^ (mg/kg)	Mg^2+^ (mg/kg)
ST 1								
Mean	0.13 ± 0.031a	2.53 ± 0.427a	5.15 ± 0.375a	17.92 ± 0.699a	163.11 ± 39.138a	48.95 ± 7.641a	1695.96 ± 820.095a	21.71 ± 3.646a
Range	0.11–0.16	2.25–3.02	4.78–5.53	17.52–18.73	117.97–187.52	41.74–56.96	773.53–2342.65	17.59–23.04
ST 2								
Mean	0.12 ± 0.004a	2.67 ± 0.332a	5.31 ± 0.112a	15.34 ± 3.352a	178.89 ± 0.944ab	52.8 ± 5.119a	1487.69 ± 550.722a	22.01 ± 0.577a
Range	0.11–0.13	2.28–2.89	5.22–5.43	11.48–17.52	178.28–179.98	47.4–57.58	1081.62–2114.55	21.41–22.56
ST 3								
Mean	0.14 ± 0.059a	4.08 ± 1.827a	5.80 ± 0.958a	18.73 ± 5.539a	173.42 ± 30.609ab	44.86 ± 3.373a	1903.18 ± 618.018a	23.14 ± 1.141a
Range	0.11–0.21	2.66–6.14	5.08–6.89	13.89–24.77	142.02–203.17	42.41–48.71	1208.82–2574.26	22.07–24.34
ST 4								
Mean	0.09 ± 0.016a	2.39 ± 0.530a	5.46 ± 0.285a	15.91 ± 3.045a	192.93 ± 9.793ab	57.29 ± 7.740a	2536.77 ± 802.410a	23.27 ± 0.569a
Range	0.07–0.10	1.93–2.97	5.17–5.74	12.68–18.73	183.74–203.23	48.84–64.04	1627.21–3144.49	22.83–23.91
ST 5								
Mean	0.12 ± 0.014a	2.45 ± 0.544a	5.14 ± 0.185a	18.02 ± 0.699a	209.41 ± 22.058b	73.45 ± 41.151a	2129.29 ± 674.501a	23.86 ± 1.105a
Range	0.11–0.13	1.83–2.86	4.96–5.33	17.52–18.93	185.97–229.76	48.08–120.93	1682.35–2905.15	22.65–24.82
*F*-value	1.198	1.789	0.950	0.611	1.596	0.991	0.969	0.739
*P*-value	0.37	0.208	0.475	0.644	0.25	0.456	0.466	0.586

Values are mean ± SD. For each column, values with different letters are significantly different at *P *=* *0.05.

### Effects of Masson pine mortality on forest composition and community structure

A total of 37 tree species (DBH ≥ 2.5 cm) in 30 families and 33 genera were identified in the 15 study plots (Table4). The species with the greatest number of individuals were *P. massoniana*, *Quercus aliena* Blume., *Cinnamomum camphora* (L.) Presl., and *Quercus variabilis* Blume. Only *P. massoniana* and *Q. aliena* were encountered in all sampled plots. Despite the wide variation in the stand composition of the pine ecosystem, *P. massoniana* had the highest IV (49.63%) almost 5.9 times that of the second highest IV (8.41%) observed for *C. camphora* and ranked as the dominant tree species in the study area. *Pinus massoniana* possessed the highest IV at the tree layer, followed by *C. camphora*, *Q. aliena*, *Q. variabilis*, and *Loropetalum chinensis* (R. Br.) Oliver. The remaining species included *Koelreuteria paniculata* Laxm., *Broussonetia papyrifera* (L.) Vent., *Elaeocarpus sylvestris* (Lour.) Poir., and nine other species that appeared in fewer plots and had lower IV.

**Table 4:** Important tree species with importance value (IV) over 0.50 in Masson pine forest

Species	Number of individuals	Number of plots	Total basal area (m^2^)	DBH (cm)	Height (m)	Crown breadth (m^2^)	Importance value (%)
*Pinus massoniana*	1187	15	22.82	12.89 ± 8.90	10.41 ± 2.61	16.92 ± 16.61	49.63
*Cinnamomum camphora*	160	9	3.29	13.89 ± 9.81	9.12 ± 2.37	28.82 ± 13.45	8.41
*Quercus aliena *	202	15	0.75	5.85 ± 3.59	5.35 ± 2.43	10.81 ± 10.22	7.39
*Quercus variabilis*	116	13	0.54	6.93 ± 3.33	5.57 ± 1.95	11.98 ± 8.58	5.3
*Loropetalum chinensis*	92	12	0.22	4.98 ± 2.39	5.69 ± 1.83	17.87 ± 10.51	4.31
*Rhus chinensis*	37	7	0.19	4.72 ± 3.14	4.25 ± 1.66	9.89 ± 13.94	2.31
*Celtis bungeana*	18	8	0.03	4.09 ± 1.43	4.36 ± 1.56	8.55 ± 5.06	2.02
*Trachycarpus fortunei*	13	6	0.08	8.64 ± 2.38	2.96 ± 1.13	12.69 ± 18.56	1.58
*Cotinus coggygria*	11	6	0.02	4.6 ± 1.35	3.69 ± 1.31	6.79 ± 4.26	1.47
*Litsea cubeba*	9	6	0.02	4.46 ± 1.65	3.56 ± 1.72	6.29 ± 2.98	1.44
*Symplocos paniculata*	11	5	0.01	3.61 ± 0.94	3.43 ± 0.84	6.65 ± 4.60	1.25
*Rhus typhina*	20	4	0.03	4.33 ± 1.35	5.61 ± 1.69	11.63 ± 7.99	1.21
*Dalbergia hupeana*	30	3	0.05	4.17 ± 1.50	3.66 ± 1.46	6.07 ± 6.76	1.19
*Ilex cornuta*	6	5	0.01	3.43 ± 0.85	2.98 ± 0.71	3.65 ± 1.84	1.17
*Albizia kalkora*	10	4	0.11	7.99 ± 7.63	5.12 ± 3.41	9.16 ± 9.12	1.14
*Symplocos caudata*	9	4	0.01	4.16 ± 1.24	3.43 ± 1.35	12.38 ± 12.27	1.01
*Aralia chinensis*	6	4	0.04	7.43 ± 6.60	7.03 ± 3.10	18.96 ± 32.82	0.99
*Rhamnus parvifolius*	18	3	0.02	3.48 ± 0.63	3.26 ± 0.90	6.97 ± 6.82	0.96
*Castanea mollissima*	5	3	0.01	3.14 ± 0.79	4.62 ± 0.79	11.52 ± 9.08	0.73
*Pistacia chinensis*	5	2	0.03	8.18 ± 4.49	4.64 ± 1.67	15.18 ± 13.16	0.54
*Deutzia grandiflora*	5	2	0.01	3.86 ± 1.01	2.93 ± 1.11	4.66 ± 1.58	0.52
*Camellia oleifera*	5	2	0.01	3.61 ± 0.67	3.68 ± 1.60	5.62 ± 4.43	0.52
*Melia azedarach*	4	2	0.01	4.65 ± 0.66	5.63 ± 0.85	5.51 ± 2.21	0.5
*Sapium sebiferum*	4	2	0.01	7.16 ± 3.23	6.01 ± 2.22	5.67 ± 2.36	0.5
*Sabina chinensis*	4	2	0.02	6.62 ± 2.01	4.63 ± 1.08	5.73 ± 2.07	0.5
Remaining species(12)	19	8	0.11	4.82 ± 2.12	4.86 ± 1.63	13.23 ± 15.09	3.41

Major community structural change occurred over the course of PWD infection and the human cutting of infected trees as *B. xylophilus* sharply decreased the prevalence of Masson pine in the forest canopy. Results for the IV of live stems (DBH ≥ 2.5 cm) at the tree layer indicated that *P. massoniana* was the most prevalent species at the control sites (ST1, IV = 67.23%), while after infection, this species showed a sharp decrease in IV (ST2–ST5, IV = 53.61–29.24%). The change in the IV of Masson pine between the control and infested stands shows that Masson pine forest communities have been significantly affected by the invasion of *B. xylophilus*. *Pinus massoniana* had the highest IV at the control sites, followed by *Q. aliena*, *R. chinensis*, and *R. parvifolius* (Table5). As the intensity of PWD damage increased, the importance of *P. massoniana* decreased at the infection sites. Conversely, the IV of broad-leaved species such as *Q. variabilis*, *L. chinensis*, *Q. aliena*, and *C. camphora* were higher at infected sites than at control sites (Table5). This result suggests that these species increased in dominance following the mortality of Masson pine trees.

**Table 5:** Importance values of Masson pine compared to other woody plant species in five Masson pine forest sites infected for different periods of time by pine wood nematode

Species	Importance value (%)				
	ST1	ST2	ST3	ST4	ST5
*Pinus massoniana*	67.23	53.61	50.24	42.65	29.24
*Cinnamomum camphora*		3.44		2.44	32.71
*Quercus aliena *	6.01	4.49	3.79	13.71	3.69
*Quercus variabilis*	2.14	3.36	10.74	5.06	
*Loropetalum chinensis*		5.06	2.18	8.18	2.44
*Rhus chinensis*	3.59	2.34	2.35		5.17
*Celtis bungeana*	2.14	2.34	2.16	2.72	2.19
*Trachycarpus fortunei*		2.42	2.58	1.89	2.35
*Cotinus coggygria*		2.04	2.27	1.87	2.54
*Litsea cubeba*	2.19	2.14	2.16	1.88	2.31
*Symplocos paniculata*	2.78				3.44
*Rhus typhina*		3.69		1.86	
*Dalbergia hupeana*			3.59		
*Ilex cornuta*	2.26	2.04	1.95		
*Albizia kalkora*	2.23		2.77		2.31
*Symplocos caudata*		2.62		1.86	
*Aralia chinensis*		1.96	1.96	1.96	2.47
*Rhamnus parvifolius*	2.59		2.75		
*Castanea mollissima*		2.21	1.95		
*Pistacia chinensis*			2.46		
*Deutzia grandiflora*	2.26		2.05		
*Camellia oleifera*				2.29	
*Melia azedarach*				2.2	
*Sapium sebiferum*				1.99	2.36
*Sabina chinensis*	2.38				
Remaining species	2.2	6.24	2.05	7.44	6.78

### Ordination of plant community structure

Based on Monte Carlo permutation with 499 iterations, five environmental variables were found to be significantly related (*P *<* *0.05) with the ordination of plant community structure through the forward selection procedure in RDA: Masson pine stumps (MPS), K^+^, capillary water holding capacity (CWHC), capillary porosity (CP), and soil water content (SWC) (Lambda-A, Table6).

**Table 6:** Marginal and conditional effects of environmental variables on plant community structure obtained from the summary of forward selection in redundancy analysis

Variables	Lambda-A^1^			Lambda-B^2^		
	Trace	*F*-ratio	*P*-value^3^	Trace	*F*-ratio	*P*-value^3^
Masson pine stumps (MPS)	0.18	2.95**	0.002	0.18	2.95**	0.002
K^+^	0.17	2.57*	0.014	0.14	2.31*	0.026
Capillary water holding capacity (CWHC)	0.14	2.13*	0.042	0.08	1.56	0.148
Capillary porosity (CP)	0.13	2.02*	0.049	0.05	0.82	0.622
Soil water content (SWC)	0.16	2.49*	0.018	0.04	0.70	0.720

1 Describes marginal effects, which show the variance explained when the variable is used as the only factor.

2 Describes conditional effects, which show the additional variance each variable explains when it is included in the model.

3 The Monte Carlo permutation with 499 iterations was performed at the 0.05 significance level.

* Significant at P < 0.05

** Significant at P < 0.01.

As shown by the RDA results in Table7 (first axis eigenvalue = 0.256 canonical, *F*-ratio = 1.721, *P *<* *0.05, 499 permutations), nearly half of the variation in plant community structure can be explained through ordination by the five selective environmental variables (sum of the trace of Lambda-B, Table6). In addition, the first two RDA axes explained 74.1% of the variance in the relationship between plant community structure and the five selective environmental variables; therefore, the analyzed relationships can be considered highly significant. Among the five factors identified in the RDA, the plant community structure was principally related to MPS (*F*-ratio = 2.95, *P *<* *0.05) and K^+^ (*F*-ratio = 2.31, *P *<* *0.05), while the other three variables did not significantly contribute to this distribution (Lambda-B, Table6). Despite its comparatively high marginal effect, SWC (*F*-ratio = 0.70, *P *>* *0.05) had no significant relationship with plant community structure.

**Table 7:** Summary of statistical results of the redundancy analysis

Canonical axes	1	2	3	4	Total variance	*F*-ratio	*P*-value
Eigenvalues	0.260	0.105	0.086	0.022	1	1.721*	0.018
Species-environment correlations	0.917	0.723	0.778	0.482			
Cumulative percentage of species variance (%)	26.0	36.5	45.1	47.3			
Cumulative percentage of species-environment relation (%)	53.2	74.6	92.2	96.7			
Sum of all eigenvalues					1		
Sum of all canonical eigenvalues					0.489		

* Significant at *P *<* *0.05.

**Figure 4:**
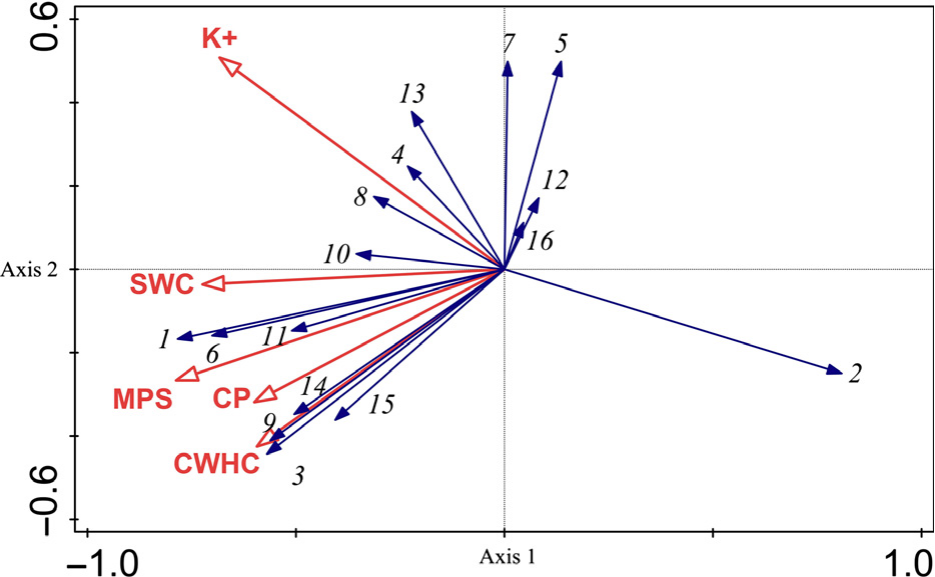
First two canonical axes of the redundancy analysis (RDA) ordination biplot between plant community structure and dominant environmental variables. MPS, Masson pine stumps; K^+^, the content of K^+^ at 0–40 cm depth; CWHC, capillary water holding capacity; CP, capillary porosity; SWC, soil water content. 1. *Cinnamomum camphora*; 2. *Pinus massoniana*; 3. *Quercus aliena*; 4. *Quercus variabilis*; 5. *Loropetalum chinensis*; 6. *Rhus chinensis*; 7. *Celtis bungeana*; 8. *Trachycarpus fortunei*; 9. *Cotinus coggygria*; 10. *Litsea cubeba*; 11. *Symplocos paniculata*; 12. *Rhus typhina*; 13.*Dalbergia hupeana*; 14. *Ilex cornuta*; 15. *Albizia kalkora*; 16. *Symplocos caudata*.

The RDA ordination biplot, with plant community structure and environmental variables along the first two axes, is shown in Fig.4. MPS, CP, and CWHC were positively associated with the growth of broad-leaved tree species *C. camphora*, *R. chinensis*, *Cotinus coggygria* Scop., *Q. aliena*, and *Albizia kalkora* (Roxb.) Prain, but were negatively associated with that of *L. chinensis*, *R. typhina*, and *Symplocos sumuntia* Buch. As revealed by the ordination graph, some species were clearly associated with higher soil water content, such as *Litsea cubeba* (Lour.) Pers., *C. camphora*, and *R. chinensis*. K^+^ was positively associated with the distribution of *Q. variabilis* and *Trachycarpus fortune* (Hook.) H. Wendl. The RDA ordination graph indicates that MPS, K^+^, and SWC were negatively associated with the growth and spatial distribution of *P. massoniana*. Simultaneously, many plant species were apparently unassociated with any of the five dominant environmental variables (Fig.3).

## Discussion

In recent years, *B. xylophilus* has seriously invaded the Three Gorges reservoir region. Many studies have demonstrated that a large disturbance, especially by an invasive plant or animal, can alter the vegetation succession pattern, damage the native ecosystem, and homogenize local biodiversity (Fujihara 1997; Fujihara et al. 2002; Conner et al. 2005). The basic conditions of the pine forest, such as stand structure, microclimate, and soil moisture and nutrient conditions, different between the control stand and the stands that had been harvested after infection with PWD. To explore the influence of PWD on the ecosystem in the Three Gorges reservoir region, this study examined the soil water-related physical and chemical properties and the plant community structure of Masson pine forest stands after infection by PWD. We determined that the stand structure was significantly different after infection and that the disturbance associated with PWD stands was important for enhancing species diversity, especially the number of deciduous species (Sakamoto et al. 2003). The recovery of vegetation and emergence of abundant woody plants causes soil property changes, such as reduced soil bulk density, that seem to increase soil aggregate stability and saturated hydraulic conductivity (Li and Shao 2006). Our study was in accordance with these previous conclusions, indicating differences in the soil properties and the composition and distribution of the plant community in Masson pine forest stands after infection by PWD.

Soil water-related physical properties are key determinants of soil fertility and soil water conservation capacity. Soil bulk density is the ratio of soil mass to the bulk or macroscopic volume of soil particles and pore spaces in a sample (Black and Hartge 1986). This value reflects soil compaction and is often used as a measure of soil structure. In the present study, the values of soil bulk density at the infected stands (ST2, ST3, ST4, and ST5) were lower than that at the control (ST1). In addition, the differences among the groups were statistically significant at the 0–10 cm and 10–20 cm soil layers (*P *<* *0.05) but were not significant at 20–40 cm (*P *>* *0.05). These results indicate that outbreaks of PWD can significantly reduce soil bulk density at the 0–20 cm soil layer, which may occur due to the greater instability of the soil structure caused by the recovery of vegetation and emergence of abundant woody plants after cutting infected trees in the Masson pine ecosystem (Li and Shao 2006).

Soil porosity, including capillary porosity (pore size < 0.1 mm) and noncapillary porosity (pore size ≥ 0.1 mm), refers to the amount of soil pores that can be filled by water or air and is closely related to soil physical behavior and root penetration (Pagliai and Vignozzi 2002; Sasal et al. 2006; Tangyuan et al. 2009). This factor is the most critical determinant of the movement of water into the soil and is closely related to soil aeration and water permeability (Diaz and Nortcliff 1985; Berger and Hager 2000). In this study, the changes of soil structure were more significant for soil total porosity and capillary porosity than for noncapillary porosity. The value of soil noncapillary porosity fluctuated, but no obvious change was observed between control and infected stands. However, the values of soil total porosity and capillary porosity were higher in the infected sands, and differences in these factors were significant among the groups (*P *<* *0.05). Greater porosity contributes to the effective infiltration of precipitation and the aeration of soil (Li and Shao 2006), benefitting the growth of plant root systems and plant community succession. Along with the greater total soil porosity at the infected sites, other soil water-related physical properties, including maximum water holding capacity, capillary water holding capacity, and soil water content, were also greater, and the differences between the stand types were significant in all soil layers (*P *<* *0.05). The differences in soil water-related physical properties according to PWD damage may be attributed to the complex responses of PWD incidence to temperature and differences in the condition of organic matter (Yoshimura et al. 1999; Kim et al. 2010; Jeong et al. 2013).

After the invasion of *B. xylophilus*, the values of soil chemical properties differed among the stand types. Total nitrogen and organic matter were slightly higher in healthy forest than in the damaged stands (Table3), despite their similarity in soil texture (Table2). These results were supported by a study conducted by Mabuhay and Nakagoshi (2012), who found that total nitrogen was higher in the top 5 cm of healthy sites undamaged by PWD than in that of infected sites. In addition, the levels of available phosphorus, sodium, potassium, calcium, and magnesium were generally slightly higher at the damaged sites than at the control sites. However, Kim et al. (2010) found that soil fertility was generally higher at undamaged sites than at damaged plots. The differences in these results may be due to the variation of soil properties with the observation period, forest conditions, and vegetation structure of the sites. Considered together, the differences of soil chemical properties were not statistically significant (*P *>* *0.05), which may be due to the short-term stability of soil nutrient element composition. The changes of soil chemical properties as the extent of infection increases are not yet well understood and require further study.

The selective cutting of Masson pine trees infected by *B. xylophilus* opened canopy gaps in the forest, which has facilitated significant changes in forest composition and community structure (Spiegel and Leege 2013). The IVs indicate that the Masson pine ecosystem is dominated by relatively few species at the tree layer. *Pinus massoniana* is the dominant species, comprising 49.63% of the total IV. Another structural characteristic of the pine ecosystem is the relatively large IVs of broad-leaved tree species such as *Q. variabilis*, *L. chinensis*, *Q. aliena*, and *C. camphora*. At sites where *B. xylophilus* caused serious damage, Masson pine mortality was significant. These canopy gaps presumably generated the community composition shift observed in comparison with less-altered sites. These results suggest that Masson pine does not become reestablished following PWD-induced mortality but is instead replaced throughout the research area by broad-leaved tree species, which has implications for shifting forest composition and community structure. As the once extremely dominant Masson pine declines, this tree's control of ecosystem structure and processes may wane (Ellison et al. 2005).

Among the 19 examined environmental variables, five were found to be significantly related (*P *<* *0.05) with the distribution of plant community structure: MPS, K^+^, CWHC, CP, and SWC (Table6). The results of RDA ordination indicated that the plant community structure was significantly related to MPS and K^+^, while the other three variables did not significantly contribute (*P *>* *0.05) to this distribution. The RDA ordination graph indicates that MPS is positively related with the growth and spatial distribution of the broad-leaved tree species *C. camphora*, *R. chinensis*, *C. coggygria*, *Q. aliena*, and *A. kalkora*. It must be noted that the five dominant environmental variables were negatively associated with the spatial distribution of *P. massoniana*, which indicates that the invasion of *B. xylophilus* suppresses the growth of this species and accelerates the succession from pine forest to broad-leaved forest.

The practice of selectively cutting dead Masson pine trees is important for preventing the spread of PWD. As the degree of damage increases, PWD results in an increasing number of stumps due to this selective cutting. The invasion of *B. xylophilus* has two general types of effects: economic and ecological. From an economic viewpoint, the loss of timber caused by PWD in China is estimated to be 5.0 × 10^6 ^m^3^, with a direct economic loss of approximately renminbi (RMB) 2.5 billion yuan and indirect economic losses exceeding RMB 25 billion yuan (Zhang and Luo 2003). The outbreak of PWD also has multiple effects on tree growth rates, plant canopy structure, and arthropods at the stand level, which in turn may alter stand development patterns such as stand structure, composition, and productivity (Veblen et al. 1991; Li et al. 2012). In addition, this disturbance can affect ecological niches in the short term, as deciduous species benefit from the release of space and resources associated with the death of the canopy-dominating pine trees (Fujihara 1996). Previous studies have also concluded that the growth of some suppressed deciduous vegetation is accelerated after PWD infection, with the dominant vegetation changing from pine to deciduous oak (Fujihara 1997; Fujihara et al. 2002). Additional work has shown that mixed forests are more stable against pests than are pure forests (Humphrey et al. 1999; Hambäck et al. 2000; Jobidon et al. 2004; Li et al. 2012).

*Bursaphelenchus xylophilus* epidemics have resulted in significant pine tree mortality across millions of hectares in central and southeastern China. Unfortunately, Masson pine is susceptible to attack by *B. xylophilus* and the main measure used to control PWD to date has been the eradication of infected pines, which is costly and only partially effective (Shi et al. 2007; Yu et al. 2011). Therefore, the unchecked spread of PWD has raised fears that Masson pine will approach functional extinction across its range. This disturbance will cause remarkable changes in forest physical conditions and soil conditions, as well as soil microbiology (Fujihara et al. 2002; Johnston and Crossley 2002). Additionally, the effects of massive Masson pine mortality may proliferate through the wood web and affect community structure (Spiegel and Leege 2013). Of even greater concern is that creatures specifically dependent on Masson pine as a natural habitat or food source may experience significant population declines. Wild life habitat and bird nesting sites may also be reduced due to the mortality of Masson pine (Osborne 1985; Spiegel and Leege 2013).

In our study, stand types were not replicated because it is impossible to find replicate Masson pine stands with the same extent of PWD infection, stand composition, soil type, and environmental conditions in two different regions (Li et al. 2011). Our results suggest that in the infected sites, Masson pine mortality significantly altered soil water-related physical properties but not chemical properties. We surveyed sites at 1–7 years after PWD was detected, which was too soon for soil chemical properties to adequately respond to weathering processes, light conditions, and changes in forest composition where Masson pine has severely declined (Kim et al. 2010; Spiegel and Leege 2013). Finally, the data gathered from the control sites may be useful for pre- and postinfection analysis (Spiegel and Leege 2013), should these sites become infected by *B. xylophilus* in the future.

In summary, our research reveals differences in the soil properties and the composition and distribution of the plant community in Masson pine forest stands after infection by PWD. In general, soil water-related physical properties differed significantly between the stand types (*P *<* *0.05), but soil chemical properties were not significantly different (*P *>* *0.05). Masson pine does not reestablish following PWD-induced mortality but is instead replaced by broad-leaved tree species. In addition, plant community composition and structure are strongly related to MPS and K^+^ in the infected plots. Given its formerly wide distribution, the extensive loss of Masson pine may have far reaching implications for the ecological security of the Three Gorges reservoir region in China. We hope to work with these sites over time to determine their long-term changes in soil properties and plant community composition and structure.
